# Resistência às drogas antituberculose na fronteira do Brasil com Paraguai e Bolívia

**DOI:** 10.26633/RPSP.2017.9

**Published:** 2017-02-08

**Authors:** Marli Marques, Eunice Atsuko Totumi Cunha, Maria do Socorro Nantua Evangelista, Paulo Cesar Basta, Ana Maria Campos Marques, Julio Croda, Sonia Maria Oliveira de Andrade

**Affiliations:** 1 Universidade Federal de Mato Grosso do Sul Programa de Pós-Graduação em Doenças Infecciosas e Parasitárias Campo Grande (MS) Brasil Universidade Federal de Mato Grosso do Sul, Programa de Pós-Graduação em Doenças Infecciosas e Parasitárias, Campo Grande (MS), Brasil.; 2 Laboratório Central de Saúde Pública de Mato Grosso do Sul Laboratório Central de Saúde Pública de Mato Grosso do Sul Campo Grande (MS) Brasil Laboratório Central de Saúde Pública de Mato Grosso do Sul, Campo Grande (MS), Brasil.; 3 Universidade de Brasília Programa de Pós-Graduação da Faculdade de Ciências da Saúde e de Enfermagem Brasília (DF) Brasil Universidade de Brasília, Programa de Pós-Graduação da Faculdade de Ciências da Saúde e de Enfermagem, Brasília (DF), Brasil.; 4 Escola Nacional de Saúde Pública (ENSP) Fundação Oswaldo Cruz (FIOCRUZ) Rio de Janeiro (RJ) Brasil Escola Nacional de Saúde Pública (ENSP), Fundação Oswaldo Cruz (FIOCRUZ), Rio de Janeiro (RJ), Brasil.; 5 Universidade Anhanguera/Uniderp Faculdade de Medicina Campo Grande (MS) Brasil Universidade Anhanguera/Uniderp, Faculdade de Medicina, Campo Grande (MS), Brasil.; 6 Universidade Federal da Grande Dourados Programa de Pós-Graduação da Faculdade de Ciências da Saúde Dourados (MS) Brasil Universidade Federal da Grande Dourados, Programa de Pós-Graduação da Faculdade de Ciências da Saúde, Dourados (MS), Brasil.

**Keywords:** Tuberculosis, pulmonary, drug resistance, border areas, diabetes mellitus, alcoholism, Brazil

## Abstract

**Objetivo.:**

Estimar as taxas de resistência às drogas entre casos de tuberculose pulmonar (TBP) para o estado de Mato Grosso do Sul, Brasil, e especificamente para a região da fronteira com Paraguai e Bolívia, além de identificar fatores de risco associados.

**Métodos.:**

O presente estudo epidemiológico, transversal, enfocou os casos de TBP registrados de janeiro de 2007 a dezembro de 2010 no Sistema de Informação de Agravos de Notificação da Secretaria de Estado de Saúde com resultados do teste de suscetibilidade a rifampicina, isoniazida, etambutol e estreptomicina. Definiram-se como variáveis dependentes o desenvolvimento de resistência a uma única droga e a qualquer combinação de drogas. As variáveis independentes foram ser caso novo ou tratado, residência em região de fronteira ou outra região, presença ou ausência de diabetes e história de alcoolismo.

**Resultados.:**

*Foram identificados 789 casos de TBP com teste de suscetibilidade. As características associadas à resistência foram: caso tratado (*P*=0,0001), região de fronteira (*P*=0,0142), alcoolismo (*P*=0,0451) e diabetes (*P*=0,0708). As taxas de resistência combinada, primária e adquirida no estado foram de 16,3%, 10,6% e 39,0%, e na fronteira, de 22,3%, 19,2% e 37,5%. As taxas de resistência a múltiplas drogas combinada, primária e adquirida no estado foram de 1,8%, 0,6% e 6,3%, e na fronteira, de 3,1%, 1,2% e 12,5%.*

**Conclusões.:**

O estado deve, na região de fronteira, realizar cultura em todos os sintomáticos respiratórios, investigar o padrão de resistência nos casos confirmados, adotar o tratamento diretamente observado nos casos de TBP e desencadear ações de saúde conjuntas com os países fronteiriços. Em todo o estado, é necessário monitorar os níveis de resistência adquirida, ampliar a investigação de resistência para todos os casos tratados e adotar o tratamento diretamente observado prioritariamente entre pacientes com alcoolismo e diabetes.

## Abstract (en)

**Objective.:**

To estimate the rate of drug resistance among pulmonary tuberculosis (PTB) cases in the state of Mato Grosso do Sul, Brazil, and specifically in the border areas with Paraguay and Bolivia, as well as to identify associated risk factors.

**Method.:**

The present cross-sectional, epidemiological study focused on PTB cases recorded between January 2007 and December 2010 in the State Reportable Disease Information System with results of susceptibility tests to rifampicin, isoniazid, ethambutol, and streptomycin. Dependent variables were development of resistance to a single drug or any combination of drugs. Independent variables were being a new or treated case, living in border areas, presence/absence of diabetes, and history of alcoholism.

**Results.:**

There were 789 TBP cases with susceptibility testing. The following characteristics were associated with resistance: treated case (*P*=0.0001), border region (*P*=0.0142), alcoholism (*P*=0.0451), and diabetes (*P*=0.0708). The rates of combined, primary, and acquired resistance for the state were 16.3%, 10.6%, and 39.0%, vs. 22.3%, 19.2%, and 37.5% for the border region. The rates of combined, primary, and acquired multidrug resistance for the state were 1.8%, 0.6%, and 6.3%, vs. 3.1%, 1.2%, and 12.5% for the border region.

**Conclusions.:**

In the border region, the state should investigate drug resistance in all patients with respiratory symptoms, determine the pattern of resistance in confirmed cases, adopt directly observed treatment for cases of PTB, and develop health actions together with neighboring countries. Across the state, the levels of acquired resistance should be monitored, with investigation of resistance in all treated cases and implementation of directly observed treatment especially among patients with diabetes or alcoholism.

A história natural da tuberculose se transformou a partir da era quimioterápica, marcada pela descoberta da estreptomicina em 1943 e da rifampicina em 1963. Desse momento em diante, a tuberculose deixou de ser uma doença devastadora para se tornar uma doença curável. No entanto, com a expansão do uso da rifampicina após 1979, identificaram-se cepas de *Mycobacterium tuberculosis* resistentes a essa droga entre pessoas com tuberculose que haviam apresentado resistência a isoniazida (1, 2).

A resistência simultânea a rifampicina e isoniazida, definida como resistência a múltiplas drogas (*multidrug resistance,* MDR) (3, 4), causou dificuldades adicionais para o controle da tuberculose, especialmente nos países em desenvolvimento. A tuberculose resistente a múltiplas drogas (TB-MDR) não responde ao tratamento padrão com 6 meses de antibacilares de primeira linha, requerendo a utilização de outros fármacos mais tóxicos, dispendiosos e menos eficazes, aumentando, assim, o tempo de tratamento (5-8).

Entre 1994 e 2007, ocorreu um aumento significativo das taxas de TB-MDR em nível mundial, principalmente entre casos novos, indicando transmissão recente. Níveis alarmantes foram detectados em 14 países da antiga União Soviética e quatro províncias da China (4). Nessas regiões, as taxas de MDR oscilaram entre 6% e 23%; além disso, houve registro de casos de tuberculose extensivamente resistentes (TB-XDR), que resultam do tratamento malsucedido dos doentes resistentes a múltiplas drogas e que desenvolveram, adicionalmente, resistência a uma das drogas injetáveis de segunda linha (fluoroquinolonas), como a capreomicina, canamicina ou amicacina (8, 9).

Segundo a Organização Mundial da Saúde (OMS), em 2012 foram confirmados 83 715 casos de TB-MDR, sendo 43,8% na Europa, 23,0% na Ásia, 5,3% no Pacífico Sul, 4,8% nas Américas e 2,7% no Mediterrâneo (9). A despeito de as Américas não apresentarem as maiores taxas, a TB-MDR vem se expandindo na América do Sul (10).

No Brasil, dados preliminares do II Inquérito Nacional de Resistência aos Fármacos Antituberculose, realizado entre 2006 e 2008 com 4 421 pacientes de sete unidades da federação, revelaram taxas de 1,4% para MDR primária (verificada em pacientes nunca tratados para tuberculose) e de 7,5% para MDR adquirida (verificada em pacientes que se tornaram resistentes após exposição ao tratamento da tuberculose) (3, 4, 11). No estado de Mato Grosso do Sul, entre 2001 e 2006, considerando-se todas as formas de tuberculose, a taxa de MDR combinada foi de 4,9%, assim distribuída: MDR primária de 1,6%; MDR adquirida de 20,3%, esta última muito mais alta do que aquela observada para o Brasil (12). No Paraguai e na Bolívia, países limítrofes ao estado de Mato Grosso do Sul, no Brasil, as taxas registradas de MDR adquirida chegam a 14,6% e 16,0%, respectivamente (10).

A taxa de incidência da tuberculose pulmonar (TBP) estimada para o Brasil e o estado de Mato Grosso do Sul como um todo são similares. No entanto, essa taxa é mais elevada em alguns segmentos populacionais e regiões de Mato Grosso do Sul. Entre 2007 e 2010, o estado registrou taxa de incidência de TBP de 31,5/100 mil habitantes, taxa de mortalidade de 2,3/100 mil habitantes e taxa de abandono de tratamento de 8,7%. Na região da fronteira com Paraguai e Bolívia, a incidência foi 49,1/100 mil habitantes, a mortalidade foi 4,0/100 mil habitantes e o abandono do tratamento alcançou 11,3% (13). Na população indígena da fronteira, a taxa de incidência foi de 253,4/100 mil habitantes, a mortalidade foi de 11,6/100 mil habitantes e o percentual de abandono foi de 5,3%, superando em 6,3 vezes a incidência observada para a população não indígena dessa região, e em 3,2 vezes a mortalidade (13). Esses dados evidenciam uma área crítica e um grupo populacional de maior vulnerabilidade para o controle da doença.

Considerando-se esse cenário epidemiológico, nosso objetivo foi estimar as taxas de resistência aos fármacos antituberculose entre casos de tuberculose pulmonar para o estado de Mato Grosso do Sul como um todo e para a região de fronteira, além de identificar fatores de risco associados.

## MATERIAIS E MÉTODOS

O presente estudo epidemiológico, transversal, enfocou um universo de pacientes notificados como portadores de TBP no período de janeiro de 2007 a dezembro de 2010 no estado de Mato Grosso do Sul, Brasil. O estado de Mato Grosso do Sul faz fronteira com a Bolívia e o Paraguai ao longo de 1 578 km. Nessa extensão, denominada neste estudo de região de fronteira, se localizam 12 municípios. O censo demográfico de 2010 contabilizou para o estado uma população de 2 449 024 habitantes, 12,5% residentes em municípios fronteiriços e 87,5% residentes nos demais municípios (14). Na região de fronteira, a população residente era de 305 953 habitantes, com 13 524 (4,4%) pessoas que se autodeclararam indígenas (13, 14).

Foram estudados os casos de TBP registrados no Sistema de Informação de Agravos de Notificação da Secretaria de Estado de Saúde de Mato Grosso do Sul (SINAN/SES/MS) em setembro de 2012 com resultado do teste de suscetibilidade às drogas antituberculose disponível no Laboratório Central de Saúde Pública (LACEN/MS). No LACEN/MS, as culturas identificadas como positivas para *M. tuberculosis* a partir de análise macroscópica, teste de inibição de crescimento em ácido p-nitrobenzoico (9) e teste de resistência a hidrazida do ácido tiofeno-2-carboxílico (10) foram submetidas ao teste de suscetibilidade às drogas (TSD) pelo sistema radiométrico (BACTEC MGIT 960/BD, MD, EUA). Foi testada a resistência a rifampicina, isoniazida, etambutol e estreptomicina pelo método das proporções (15). Os resultados apresentando resistência eram validados pelo Laboratório do Instituto Adolfo Lutz e, atualmente, pelo Centro de Referência Hélio Fraga no Rio de Janeiro.

No presente estudo, foram excluídos os resultados de amostras contaminadas, amostras com diagnóstico de micobactérias não tuberculosas, amostras que não contemplavam os resultados das quatro drogas, resultados em duplicata e amostras de pessoas não residentes no estado. Além disso, a base de dados foi revisada exaustivamente para evitar duplicidade de notificações e adequar-se à classificação dos casos.

### Variáveis sociodemográficas e epidemiológicas

Foram coletados os seguintes dados: sexo, idade, raça/cor, classificação do caso (caso novo, caso tratado), data de coleta do escarro, data de início do tratamento, região de residência (fronteira, não fronteira), status de institucionalização (não institucionalizado, institucionalizado em presídio, outras instituições), resultado da sorologia para o vírus da imunodeficiência adquirida (HIV) e agravos associados (diabetes e alcoolismo).

### Padrão e perfil da resistência às drogas antituberculose

Classificaram-se como “casos novos” aqueles que não apresentavam registros de notificação no SINAN/SES/MS nos 5 últimos anos e cujo teste de suscetibilidade às drogas tivesse sido realizado em amostra coletada antes de 30 dias do início do tratamento da tuberculose. “Casos tratados” foram definidos como aqueles admitidos por recidiva da doença, reingresso após abandono e aqueles que fizeram o teste de suscetibilidade às drogas no decorrer do tratamento desde que transcorridos 30 dias ou mais de uso de fármacos para tuberculose (4).

Para consolidação dos dados, utilizaram-se critérios da OMS (4), considerando os seguintes padrões de resistência: monorresistência – resistência a um fármaco antituberculose; polirresistência: resistência a dois ou mais fármacos antituberculose, exceto à associação rifampicina e isoniazida; multidrogarresistência: resistência a pelo menos rifampicina e isoniazida; resistência extensiva: resistência à rifampicina e isoniazida acrescida à resistência a uma fluoroquinolona e uma droga injetável de segunda linha (amicacina, canamicina ou capreomicina). Quanto ao perfil, definiu-se como resistência primária aquela desenvolvida a uma ou mais drogas em pacientes tratados para tuberculose por tempo inferior a 30 dias; como resistência adquirida aquela desenvolvida a uma ou mais drogas em pacientes submetidos a tratamento de tuberculose por 30 dias ou mais; e como resistência combinada a presença de resistência entre todos os casos, independentemente da história de tratamento para tuberculose (4).

### Análise estatística

Definiram-se como variáveis dependentes o desenvolvimento de resistência a uma única droga e o desenvolvimento de resistência a qualquer combinação de drogas. As variáveis independentes foram caso novo ou tratado, residência em região de fronteira ou outra região, presença ou ausência de diabetes e história de alcoolismo.

Para a tabulação e análise dos dados utilizou-se o programa Microsoft Excel, versão 7.0. Para avaliar a distribuição das variáveis, utilizou-se o teste do qui-quadrado (c^2^) para comparar proporções. O nível de significância considerado foi de *P* < 0,05. Considerando-se que as variáveis são dicotômicas, empregou-se o método de regressão logística simples para testar a associação bruta entre as variáveis dependentes e as variáveis independentes. As variáveis independentes que demonstraram associação com resistência a uma droga ou à combinação de drogas, considerando-se um nível de significância de *P* < 0,20, foram incluídas em um modelo de regressão múltipla, em ordem decrescente de valores de *P*, com base no método *stepwise forward.*

Utilizou-se como medida de associação a razão de chances (*odds ratio,* OR) e seu respectivo intervalo de confiança de 95% (IC95%). As variáveis independentes que se mantiveram associadas aos desfechos de interesse, com nível de significância de 5%, foram mantidas no modelo final.

Nesta investigação foram analisados dados secundários e em nenhum momento houve contato dos pesquisadores com os sujeitos da pesquisa. Por essa razão, não foi possível obter termos de consentimento informado individuais dos participantes. Todavia, foram seguidas recomendações da Resolução 466/12 do Conselho Nacional de Saúde (16). O estudo foi aprovado pelo Comitê de Ética em Pesquisa da Universidade Federal de Mato Grosso do Sul e pela Comissão Nacional de Ética em Pesquisa do Conselho Nacional de Saúde (CONEP/CNS) (pareceres 141.949/2012 e 507.049/2013).

## RESULTADOS

Entre janeiro de 2007 e dezembro de 2010, 3 453 casos de TBP foram notificados ao SINAN/SES/MS no estado de Mato Grosso do Sul. Entre esses casos, o TSD foi realizado em 789 (22,8%). A cobertura na região de fronteira correspondeu a 29,2% – 193 casos com TSD entre 661 casos notificados nessa região. Dentre os 789 casos do estudo, constatou-se predomínio do sexo masculino, com razão de 2,9 casos em homens para 1 caso em mulheres. A idade média foi de 39 anos, com concentração entre 30 e 64 anos (59,4%). Quanto à raça/cor, os pardos representaram 34,9%, os brancos 30,2%, os indígenas 19,4% e os pretos e amarelos menos de 10,0%. Os casos novos contabilizaram 80,0% (630/789), os residentes em fronteira contabilizaram 25,0% (193/789) e dentre os casos com informação sobre local de permanência, 17,3% (134/774) se encontravam em presídios. Quanto às comorbidades, 6,8% tinham sorologia positiva para HIV (54/789), 5,6% apresentavam diabetes (44/789) e 12,2% apresentavam alcoolismo (96/789). Detectou-se suscetibilidade às quatro drogas em 83,7% (660/789) dos casos, com presença de qualquer padrão de resistência em 16,3% (129/789).

As variáveis sociodemográficas e clínicoepidemiológicas associadas à resistência na análise bivariada foram: tratamento anterior para tuberculose (*P* = 0,0001), residir na fronteira (*P* = 0,0142), comorbidade com alcoolismo (*P* = 0,0451) e diabetes (*P* = 0,0708) (tabela 1).

A taxa de resistência encontrada entre casos novos foi de 10,6% (67/630); entre casos tratados, a taxa de resistência foi de 39,0% (62/159), superando em mais de 3 vezes a taxa para os casos novos (*P* < 0,0001). A isoniazida foi a droga com maior taxa de resistência adquirida, 12,6% (20/159). A taxa de TB-MDR combinada foi de 1,8% (14/789), a primária de 0,6% (4/630) e a adquirida de 6,3% (10/159). Não houve nenhum diagnóstico de TB-XDR. A taxa de resistência adquirida de 39,0% (62/159) foi significativamente superior à taxa de resistência primária (*P* < 0,0001). Independentemente da droga e do padrão de resistência, as taxas de resistência adquirida superaram significativamente as de resistência primária (tabela 2).

Na análise multivariada, as variáveis associadas a monorresistência e qualquer padrão de resistência foram: não ser caso novo, residência na fronteira, comorbidade com diabetes e alcoolismo (tabela 3). A distribuição dos casos segundo regiões mostrou que 24,5% (193/789) residiam na fronteira e 75,5% (596/789) nos demais municípios. Comparando as taxas de resistência combinada, resistência adquirida e resistência primária entre as duas regiões, constatou-se não haver diferença estatisticamente significativa entre os grupos de resistência combinada (*P =* 0,055) e adquirida (*P* = 0,251). Na resistência primária houve diferença significativa (*P =* 0,001) entre a fronteira e os demais municípios exclusivamente para a monorresistência (tabela 4).

Dentre os 14 casos com MDR combinada, 3,1% (6/193) residiam na fronteira, nos municípios de Corumbá e Aral Moreira, e 1,3% (8/596) nos demais municípios. Esses 14 casos tinham entre 23 e 80 anos, seis (42,8%) tinham menos de 40 anos e um apresentava comorbidade com alcoolismo.

## DISCUSSÃO

Desde 1999, o LACEN/MS vem capacitando e apoiando técnica e logisticamente os laboratórios municipais do estado para descentralizar a coleta de amostras de escarro e ampliar o diagnóstico de TBP em populações vulneráveis. A partir de 2007, esse esforço resultou na descentralização das coletas para Corumbá (fronteira com a Bolívia) e Ponta Porã (fronteira com o Paraguai), o que possibilitou a investigação de resistência em aproximadamente 30% dos casos de TBP (17) em região de elevado abandono e alto risco de adoecimento e óbito por tuberculose. Ainda assim, a taxa de qualquer resistência combinada detectada no presente estudo foi semelhante à registrada na Região das Américas (16,7%) e menor do que os 28,3% registrados na Bolívia (4).

**TABELA 1.** Características sociodemográficas, epidemiológicas e clínicas dos casos de tuberculose pulmonar segundo teste de suscetibilidade às drogas, Mato Grosso do Sul, Brasil, 2007 a 2010

Variável	Casos suscetíveis (n=660; 83,7%)		Casos resistentes (n=129; 16,3%)		*P* ^a^	Total^b^	
	No.	(%)	No.	(%)		No.	%
Sexo					0.228		
Masculino	485	73,5	102	85,7		587	74,4
Feminino	175	26,5	27	14,3		202	25,6
Idade (anos)					0.2364		
< 15	10	1,5	1	0,8		11	1,4
15–29	205	31,3	35	27,3		240	30,7
30–64	378	57,7	86	67,2		464	59,3
> 65	62	9,5	6	4,7		68	8,7
Raça/cor					0.2337		
Branca	203	30,8	35	27,1		238	30,2
Preta	48	7,3	11	8,5		59	7,5
Amarela	11	1,7	5	3,9		16	2,0
Parda	229	34,7	46	35,7		275	34,9
Indígena	134	20,3	19	14,7		153	19,4
Ignorada	35	5,3	13	10,1		48	6,1
Classificação do caso					0.0001		
Caso novo	563	85,3	67	51,9		630	79,8
Caso tratado	97	14,7	62	48,1		159	20,2
Região de residência					0.0142		
Fronteira	150	22,7	43	33,3		193	24,5
Não fronteira	510	77,3	86	66,7		596	75,5
Institucionalização					0.2320		
Não	458	70,9	80	62,5		538	69,5
Presídio	107	16,6	27	21,1		134	17,3
Outros locais	81	12,5	21	16,4		102	13,2
Resultado do HIV					0.3085		
Positivo	42	6,4	12	9,3		54	6,8
Negativo ou não realizado	618	93,6	117	90,7		735	93,2
Agravo associado-diabetes					0.0708		
Sim	32	4,8	12	9,3		44	5,6
Não ou ignorado	628	95,2	117	90,7		745	94,4
Agravo associado-alcoolismo					0.0451		
Sim	73	11,1	23	17,8		96	12,2
Não ou ignorado	587	88,9	106	82,2		693	87,8

a Análise realizada usando o χ^2^ para comparar proporções ou teste de Fisher.

b As porcentagens se referem ao total de 789 casos, exceto para idade e institucionalização.

Comparando os resultados deste estudo com as taxas de qualquer resistência primária e adquirida e de MDR primária e adquirida encontradas em Mato Grosso do Sul entre 2001 e 2006 para tuberculoses pulmonares e extrapulmonares (12), constata-se declínio exclusivamente para a taxa de MDR adquirida, passando de 20,3% para 6,3% (*P* < 0,001). Ainda, a despeito do incremento na investigação de casos tratados, que passou de 17,6% entre 2000 e 2006 (12) para 20,0% no estudo atual, e da ampliação da investigação na região de fronteira (13), a taxa de TB-MDR adquirida declinou, seguramente em decorrência do maior investimento na estruturação dos serviços de saúde e maior atenção aos doentes, com vistas a adesão e uso correto do tratamento (9, 18, 19).

A distribuição por faixa etária evidenciou casos entre menores de 15 anos e acima de 65 anos. A resistência entre jovens é atribuída a transmissão recente, visto que a idade, segundo a OMS, é um fator de risco para a resistência (9), além de refletir indiretamente a efetividade da vigilância da tuberculose no ambiente em que o jovem vive. Em Manaus, no estado do Amazonas, Garrido et al. (20) encontraram associação significativa entre resistência primária em menores de 15 anos e contato de tuberculose no domicílio, com o dobro de risco quando o contato era conhecido, fato que explica a ocorrência de resistência primária a isoniazida em criança com 11 anos. Além disso, pacientes jovens infectados pelo *M. tuberculosis* apresentam maior risco de reativação endógena frente a comorbidades, ou de evolução de infecção recente para tuberculose primária (21). Quanto à possibilidade de avaliar a taxa de transmissão ativa dessas cepas entre jovens (22), verificou-se que 50% (16/32) daqueles com resistência a estreptomicina tinham idade entre 20 e 30 anos. Nesse grupo, 37,5% (6/16) tinham comorbidade com alcoolismo ou diabetes.

A resistência a estreptomicina permite também estimar a proporção de reativação da tuberculose nos pacientes idosos, decorrente de linhagens que surgiram antes da década de 1980, visto que esse fármaco não é utilizado no Brasil entre casos novos (22). A taxa encontrada de 6,8% de resistência à estreptomicina, sozinha ou associada a outras drogas, representa uma ameaça ao controle da tuberculose em nosso meio, já que todos os tratamentos alternativos para resistência a rifampicina ou isoniazida utilizam a estreptomicina como droga de escolha (21). A resistência às drogas antituberculosas em nosso estudo mostrou-se associada a casos tratados, residência na fronteira e comorbidade com alcoolismo e diabetes.

**TABELA 2.** Taxa de resistência combinada, primária e adquirida às drogas antituberculose e padrão de resistência, Mato Grosso do Sul, Brasil, 2007 a 2010

Variável	Casos novos (n = 630)		Casos tratados (n=159)		*P*^a^	Total de casos (n=789)	
	Resistência primária		Resistência adquirida			Resistência combinada	
	No.	%	No.	%		No.	%
Sensíveis	563	89,4	97	61,0	< 0,0001	660	83,7
Qualquer resistência^b^	67	10,6	62	39,0		129	16,3
I	17	2,7	20	12,6	< 0,0001	37	4,7
S	32	5,1	15	9,4	0,002	47	6,0
E	0	0	1	0,6	0,016	1	0,1
R	4	0,6	4	2,5	0,006	8	1,0
R + I (MDR)	3	0,5	8	5,0		11	1,4
R + I + S (MDR)	1	0,2	2	1,3	< 0,0001	3	0,4
I + S	8	1,3	8	5,0	0,001	16	2,0
R + S	0	0	1	0,6		1	0,1
I + E	0	0	1	0,6		1	0,1
R + E	0	0	1	0,6		1	0,1
R + S + E	1	0,2	0	0,0		1	0,1
I + S + E	1	0,2	1	0,6		2	0,3

a χ^2^ de Fisher.

b I: isoniazida; S: estreptomicina; E: etambutol; R: rifampicina.

***Fonte:*** Sistema de Informação de Agravos de Notificação (SINAN) e Laboratório Central de Saúde Pública (LACEN), Secretaria de Estado de Saúde de Mato Grosso do Sul (SES/MS).

**TABELA 3.** Modelo multivariado de regressão logística para identificar fatores associados ao desenvolvimento de resistência considerando padrão de resistência às drogas antituberculose, Mato Grosso do Sul, Brasil, 2007 a 2010

Variável	Monorresistência						
	No.	%	OR bruta	IC95%	OR ajustada	IC95%
Caso novo	Sim	630	79,8				
	Não	159	20,2	4,4	2,8 a 7,0	4,9	3,0 a 7,9
	Total	789					
Fronteira	Sim	124	25,8	1,5	1,0 a 2,5	1,9	1,1 a 3,1
	Não	356	74,2				
	Total	480					
Diabetes	Sim	38	25,8	2,6	1,3 a 5,4	3,0	1,4 a 6,4
	Não	442	74,2				
	Total	480					
Alcoolismo	Sim	82	25,8	1,7	0,9 a 3,0	2,1	1,1 a 3,9
	Não	398	74,2				
	Total	480					
Qualquer resistência (monorresistência, MDR, polirresistência)							
		No.	%	OR bruta	IC95%	OR ajustada	IC95%
Caso novo	Sim	630	79,8				
	Não	159	20,2	5,4	3,6 a 8,1	6,0	3,9 a 9,1
	Total	789					
Fronteira	Sim	193	24,5	1,7	1,1 a 2,6	2,1	1,3 a 3,2
	Não	596	75,5				
	Total	789					
Diabetes	Sim	44	8,9	2,0	1,0 a 4,0	2,4	1,2 a 5,1
	Não	448	91,1				
	Total	492					
Alcoolismo	Sim	96	19,4	1,7	1,0 a 2,9	2,1	1,2 a 3,6
	Não	399	80,6				
	Total	495					

***Fonte:*** Sistema de Informação de Agravos de Notificação (SINAN) e Laboratório Central de Saúde Pública (LACEN), Secretaria de Estado de Saúde de Mato Grosso do Sul (SES/MS).

A associação entre resistência e casos tratados possivelmente está refletindo interrupção do tratamento, demora no diagnóstico, contatos intradomiciliares representando fonte de infecção, uso inadequado da quimioterapia, gestão inadequada pela equipe médica, precárias condições socioeconômicas e ausência de supervisão do tratamento (9). A maior suscetibilidade dos residentes na fronteira fronteira pode estar evidenciando escassos recursos laboratoriais e precário controle da tuberculose, conforme relatado para municípios amazonenses da fronteira com Colômbia-Peru-Venezuela (23) e para a população indígena da fronteira com Colômbia e Venezuela (24). Nos municípios da fronteira brasileira, ficou evidente a associação entre reingresso e abandono do tratamento, onde o abandono prévio representou 3 vezes mais chance de não conclusão do tratamento quando comparado aos casos novos, além da relevância das baciloscopias de controle na redução do abandono em quase 12 vezes entre os doentes adequadamente acompanhados (23).

**TABELA 4.** Distribuição dos casos de tuberculose pulmonar segundo perfil e padrão de resistência às drogas antituberculose e localidade, Mato Grosso do Sul, Brasil, 2007 a 2010

Perfil (padrão de resistência)	Fronteira^a^		Não fronteira^a^		Total		χ^2^	P
	No.	%	No.	%	No.	%		
Casos novos (resistência primária)								
Sensível	130	^b^80,7	433	^a^92,3	563	89,4	17,04	0,001
Monorresistência	24	^a^14,9	29	^b^6,2	53	8,4		
Polirresistência	5	^a^3,1	5	^a^1,1	10	1,6		
MDR	2	^a^1,2	2	^a^0,4	4	0,6		
Total	161		469		630			
Casos tratados (resistência adquirida)								
Sensível	20	62,5	77	60,6	97	61,0	4,1	0,251
Monorresistência	5	15,6	35	27,6	40	25,2		
Polirresistência	3	9,4	9	7,1	12	7,5		
MDR	4	12,5	6	4,7	10	6,3		
Total	32		127		159			
Total de casos (resistência combinada)								
Sensível	150	77,7	510	85,6	660	83,6	7,6	0,055
Monorresistência	29	15,1	64	10,7	93	11,8		
Polirresistência	8	4,1	14	2,4	22	2,8		
MDR	6	3,1	8	1,3	14	1,8		
Total	193		596		789			

a Letras diferentes na linha indicam diferença significativa entre fronteira e não fronteira em relação ao percentual de casos (teste do χ^2^ com correção de Bonferroni).

***Fonte:*** Sistema de Informação de Agravos de Notificação (SINAN) e Laboratório Central de Saúde Pública (LACEN), Secretaria de Estado de Saúde de Mato Grosso do Sul (SES/MS).

A forte associação entre tuberculose e uso pesado/distúrbios atribuídos ao uso abusivo de álcool foi profundamente discutida na revisão sistemática realizada por Rehm et al. (25). Esses autores destacam o impacto patogênico do álcool no sistema imunológico, causando suscetibilidade à doença além da forte influência na incidência, no prognóstico, na evolução do tratamento e na farmacocinética das drogas. A marginalização social vivenciada pelo usuário, maior taxa de reinfecção e de inadimplência ou recusas de tratamento favorecem o desenvolvimento de formas resistentes (21, 25).

No Brasil, entre 2001 e 2011 se observou aumento substancial e progressivo dos casos de tuberculose entre portadores de diabetes (26). A OMS (27) estima que até 2030 os casos de tuberculose entre pacientes com diabetes sejam triplicados devido às mudanças no padrão alimentar, estilo de vida e obesidade (28). Esse aumento será mais significativo nos países em desenvolvimento, onde a carga da tuberculose é maior e onde ocorrerão 80,0% dos casos de diabetes (27), representando uma ameaça ao controle da tuberculose (26-29). O mecanismo pelo qual os pacientes com diabetes desenvolvem resistência ainda precisa ser esclarecido (30), mas suspeita-se que decorra da progressão rápida da doença, da demora no diagnóstico da tuberculose (29, 31, 32) ou da má absorção medicamentosa, gerando baixa concentração da medicação (29).

Algumas limitações desta investigação são reconhecidas, entre elas a perda aproximada de 21,0% entre cultura positiva e realização do teste de suscetibilidade às drogas. Tais dificuldades decorrem da insuficiência de técnicos no LACEN/MS, para repetir, sempre que recomendado, os procedimentos necessários até a conclusão da investigação da resistência, além da falta de um sistema informatizado que facilite o controle dos exames a serem repetidos. Ainda assim, este universo de casos avaliados representa uma amostra de conveniência (não randomizada), que incluiu pacientes atendidos em diferentes níveis de atenção a saúde, distribuídos em cerca de 83,0% dos municípios sul-matogrossenses, em um período de 4 anos; representa ainda, provavelmente, uma amostra de pacientes com maior risco de resistência, ou de localidades e serviços com profissionais sensibilizados para o problema da resistência. Além disso, o uso de duas bases de dados secundários (SINAN/SES/MS e LACEN/MS), com possíveis inconsistências entre registros e de um universo de casos não estratificado, reflete as características do universo avaliado. Portanto, os resultados devem ser interpretados com cautela, não devendo ser extrapolados para toda a população. Apesar disso, consideramos esta análise de interesse para a gestão local, regional, estadual e países limítrofes, incluindo os profissionais que trabalham no controle da TBP.

Assim sendo, os resultados encontrados assinalam que: 1) a taxa de resistência para qualquer droga, entre casos tratados, supera as estimativas para o Brasil, muito embora a MDR primária e adquirida sejam similares às estimativas para o país, porém inferiores à registrada nas Américas; 2) a resistência às drogas antituberculosas mostrou-se associada ao tratamento prévio, morar na fronteira e ter comorbidade com alcoolismo e diabetes; 3) a taxa de resistência adquirida superou a taxa de resistência primária e foi estatisticamente significativa para qualquer droga isolada, bem como para qualquer padrão de associação de drogas; 4) dentre as variáveis de risco para resistência, não ser caso novo associou-se a chance 6,0 vezes maior de desenvolver resistência e 4,9 vezes maior de monorresistência em comparação a ser caso novo, morar na fronteira representou chance 2,1 vezes maior de desenvolver resistência e 1,9 vez maior de monorresistência em comparação a residir fora da fronteira, comorbidade com diabetes representou chance 2,4 vezes maior de desenvolver resistência e 3,0 vezes mais chance de monorresistência em comparação a ausência de diabetes, comorbidade com alcoolismo representou chance 2,1 vezes maior de desenvolver qualquer padrão de resistência em relação a ausência de alcoolismo; 5) os casos novos de TBP que residiam na região da fronteira apresentaram maior chance de adoecerem a partir de cepas resistentes a uma droga quando comparados com residentes fora de área de fronteira; 6) os doentes de TBP residentes no estado, independentemente da região, apresentaram igual chance de adquirirem resistência às drogas.

Assim, conclui-se ser necessário, para a região de fronteira, realizar cultura em todos os sintomáticos respiratórios, investigar o padrão de resistência em todos os casos confirmados, adotar o tratamento diretamente observado em todos os casos de TBP e desencadear ações de saúde conjuntas com os países fronteiriços. Para o estado como um todo, é necessário monitorar os níveis de resistência adquirida, ampliando a investigação de resistência para todos os casos tratados e adotando o tratamento diretamente observado prioritariamente entre pacientes com alcoolismo e diabetes. Também se faz necessária a prevenção do adoecimento por TBP dos portadores de diabetes e alcoolismo a fim de reduzir a carga da doença; para tanto, é necessário trabalhar de forma integrada com os respectivos programas de controle, seguindo os protocolos estabelecidos para prevenção da tuberculose.

### Agradecimentos.

Os autores agradecem às equipes de vigilância da tuberculose dos municípios sul-mato-grossenses que alimentam o SINAN, à equipe do LACEN/MS que processou os exames e disponibilizou o banco de dados e à Coordenação Nacional do Programa de Controle da Tuberculose/Ministério da Saúde pela orientação na busca de financiamento. Ao Fundo Nacional de Saúde pelo aporte financeiro (Termo de Cooperação Técnica Nº 120/2010).

### Declaração de responsabilidade.

A responsabilidade pelas opiniões expressas neste manuscrito é estritamente dos autores e não reflete necessariamente as opiniões ou políticas da *RPSP/PAJPH* nem da OPAS.
